# No fry zones: How restaurant distribution and abundance influence avian communities in the Phoenix, AZ metropolitan area

**DOI:** 10.1371/journal.pone.0269334

**Published:** 2022-10-19

**Authors:** Jeffrey A. Brown, Susannah B. Lerman, Anthony J. Basile, Heather L. Bateman, Pierre Deviche, Paige S. Warren, Karen L. Sweazea

**Affiliations:** 1 Global Institute of Sustainability, Arizona State University, Tempe, AZ, United States of America; 2 USDA Forest Service, Northern Research Station, Amherst, Massachusetts, United States of America; 3 School of Life Sciences, Arizona State University, Tempe, AZ, United States of America; 4 College of Integrative Sciences and Arts, Arizona State University, Mesa, AZ, United States of America; 5 Department of Environmental Conservation, University of Massachusetts Amherst, Amherst, Massachusetts, United States of America; 6 College of Health Solution, Arizona State University, Phoenix, AZ, United States of America; Universidade Federal de Goias, BRAZIL

## Abstract

Urbanization is one of the most widespread and extreme examples of habitat alteration. As humans dominate landscapes, they introduce novel elements into environments, including artificial light, noise pollution, and anthropogenic food sources. One understudied form of anthropogenic food is refuse from restaurants, which can alter wildlife populations and, in turn, entire wildlife communities by providing a novel and stable food source. Using data from the Maricopa Association of Governments and the Central Arizona-Phoenix Long Term Ecological Research (CAP LTER) project, we investigated whether and how the distribution of restaurants influences avian communities. The research aimed to identify restaurants, and thus the associated food they may provide, as the driver of potential patterns by controlling for other influences of urbanization, including land cover and the total number of businesses. Using generalized linear mixed models, we tested whether the number of restaurants within 1 km of bird monitoring locations predict avian community richness and abundance and individual species abundance and occurrence patterns. Results indicate that restaurants may decrease avian species diversity and increase overall abundance. Additionally, restaurants may be a significant predictor of the overall abundance of urban-exploiting species, including rock pigeon (*Columba livia*), mourning dove *(Zenaida macroura)*, and Inca dove (*Columbina Inca*). Understanding how birds utilize anthropogenic food sources can inform possible conservation or wildlife management practices. As this study highlights only correlations, we suggest further experimental work to address the physiological ramifications of consuming anthropogenic foods provided by restaurants and studies to quantify how frequently anthropogenic food sources are used compared to naturally occurring sources.

## Introduction

The Anthropocene is characterized by the global impact of humans on climate and the environment [1–3]. One of the most prominent effects is the development and conversion of land into urban and urbanizing areas [4, 5], which house most of the human population [6]. Paradoxically, urban areas can also support high levels of biodiversity, likely due to the environmental opportunities humans create [7–9]. Examples of these opportunities for urban wildlife include species introductions, alterations to habitat structure, and food subsidies [10–12]. Anthropogenic foods, defined as either intentional feeding via wildlife feeders or accidental feeding from trash spillover and litter, can shift animals’ diet and foraging strategies [13–15]. An important first step to understanding how wildlife will respond to future urbanization and why some species thrive in these developed environments is to focus on how particular features of the urban environment, such as anthropogenic food sources, influence urban wildlife.

Urban environments provide abundant supplemental food (i.e., any non-naturally occurring food) in the form of anthropogenic food (i.e., non-natural food provided by humans), particularly in residential landscapes [16]. These anthropogenic food sources can be a consistent part of the diet for urban wildlife. Species that successfully utilize anthropogenic food sources include omnivores with generalist diets, such as rock pigeons (*Columba livia*), that have undergone increases in abundance [17–19] and range expansion [20–23]. However, consumption of anthropogenic food can be associated with health risks (e.g., decreased body mass, impaired vasodilation; [24–26]). Despite these risks, anthropogenic food may be more stable across time and space than natural food sources, promoting a dependence on non-naturally occurring food sources [21, 27]. Anthropogenic food stability may result in biotic homogenization, the process by which wildlife communities across space become more similar to each other, as species that rely on supplemental food become more common across urban landscapes [28–31]. Due to the influences of anthropogenic food sources on wildlife health and abundance, many studies focus on intentionally introduced food sources (e.g., bird feeders or tourist food provision [17, 32, 33]). However, researchers have yet to fully explore the effects of unintentionally introduced food in urban environments [34, 35].

Research investigating the potential influence of unintentional feedings, such as refuse from restaurants, is limited, especially at community and regional scales (for species-specific examples see [34–36]). For this study, we focus on restaurants because although urban areas are highly heterogenous—differing based on age and region of the city—restaurants are ubiquitous and located throughout cities [37, 38]. Since restaurants are widely dispersed, they may act as pseudo-replicates across the landscape, allowing researchers to account for the heterogeneous nature of urban landscapes [39]. Specifically, by acting as a predictable source of anthropogenic food, food waste from restaurants may stabilize wildlife communities and attract species typically not associated with urban environments [40, 41]. Finally, restaurants may act as not only a predictable source of food but also a large one, with 37% of the 35 million metric tons of food waste in the United States coming from food services [42–44].

Predictable food sources may be important for birds as their resource needs shift throughout their life cycle (e.g., breeding, migration, or overwintering), and as resources in the environment change due to seasonality [45]. However, there is evidence that urbanization may decrease seasonal variability in the resources within urban environments by potentially limiting resource scarcity associated with colder seasons. Evidence of increased resource availability during winters may be seen in species overwintering in urban areas instead of migrating to areas of higher resource abundance [46, 47]. In addition to shifts from migratory to residential strategies, urbanization exerts mixed responses on avian populations, resulting in increased and decreased richness and population abundance [9, 48, 49]. By focusing on restaurants and their associated food waste, we highlight a universal feature of urban landscapes and identify their potential impact.

Using the Central Arizona-Phoenix Long-Term Ecology Research program’s (CAP LTER) avian datasets, we tested whether the presence of restaurants affects the avian abundance and species richness within the Phoenix metropolitan region while controlling for other factors associated with urbanization such as land-use and land-cover patterns as well as a general proxy for urbanization (total number of businesses). We tested three main hypotheses: (H1), the number of restaurants at a location influences avian species abundance and presence at that location; (H2), restaurants act as a predictable food source that stabilizes communities over time (i.e., avian communities near restaurants will have lower dissimilarity over time); and (H3), restaurant density influences avian community composition. Alternatively, some species may avoid other anthropogenic factors (e.g., human presence or noise), in which case there could be a negative relationship between abundance and restaurant location.

## Methods

### Study site

The Phoenix metropolitan area is a rapidly growing urban center in the state of Arizona, within the United States. Phoenix has approximately 4.7 million residents (United States Census Bureau 2019). The Phoenix metropolitan area contains 33 cities, of which Phoenix is the largest, and is comprised of primarily residential (19% landcover), urban (6% landcover), and agricultural (8% landcover) areas surrounded by undisturbed Sonoran Desert (LULC derived from [50], see below; S1 Fig). The Phoenix metropolitan landscape is home to a wide diversity of birds as it sits within a migratory flyway that connects North and South America and contains records of 316 species on eBird (as recorded on eBird.org in December of 2020 [51]).

### Bird data

The CAP LTER conducts bird surveys throughout the Phoenix metropolitan areas since 2000 [52]. Data for this work is publicly available through the CAP LTER environmental science data portal (https://data.sustainability-innovation.asu.edu/cap-portal/home.jsp). Surveys were conducted primarily in winter (January—February) and spring (April—May), with less frequent sampling in summer and fall. We only used data from winter and spring to best match consistent sampling efforts and to separate data into two distinct seasons, which reflect migratory and non-migratory bird communities. Study sites were distributed throughout the Phoenix metropolitan area to cover a broad spatial area and a diversity of land-use land-cover types. We selected bird data from 2000, 2005, and 2010 as this matched available land cover data (see below). Using multiple years provided a robust sample and allowed us to investigate how communities may change over time. A total of 57 sites were included, each sampled at least three times in the winter and three times in the spring season per year, although not all sites were sampled each year (see results for more details).

Birds were surveyed using point-count methods with an observer standing at the center of a point and recording all birds seen or heard within 40 m of the point over a 15-minute period [53]. We omitted birds flying through the point count area to ensure the species we observed were using the habitat at the point-count location. Observers recorded the number of individuals seen, the species observed, and whether the bird was seen, heard, or both seen and heard (see [52] for more details and full data). Only birds that were seen or heard and seen were included in abundance estimations as estimating abundance based on auditory measures alone can be difficult [53, 54].

### Restaurant data

**Fig 1:**
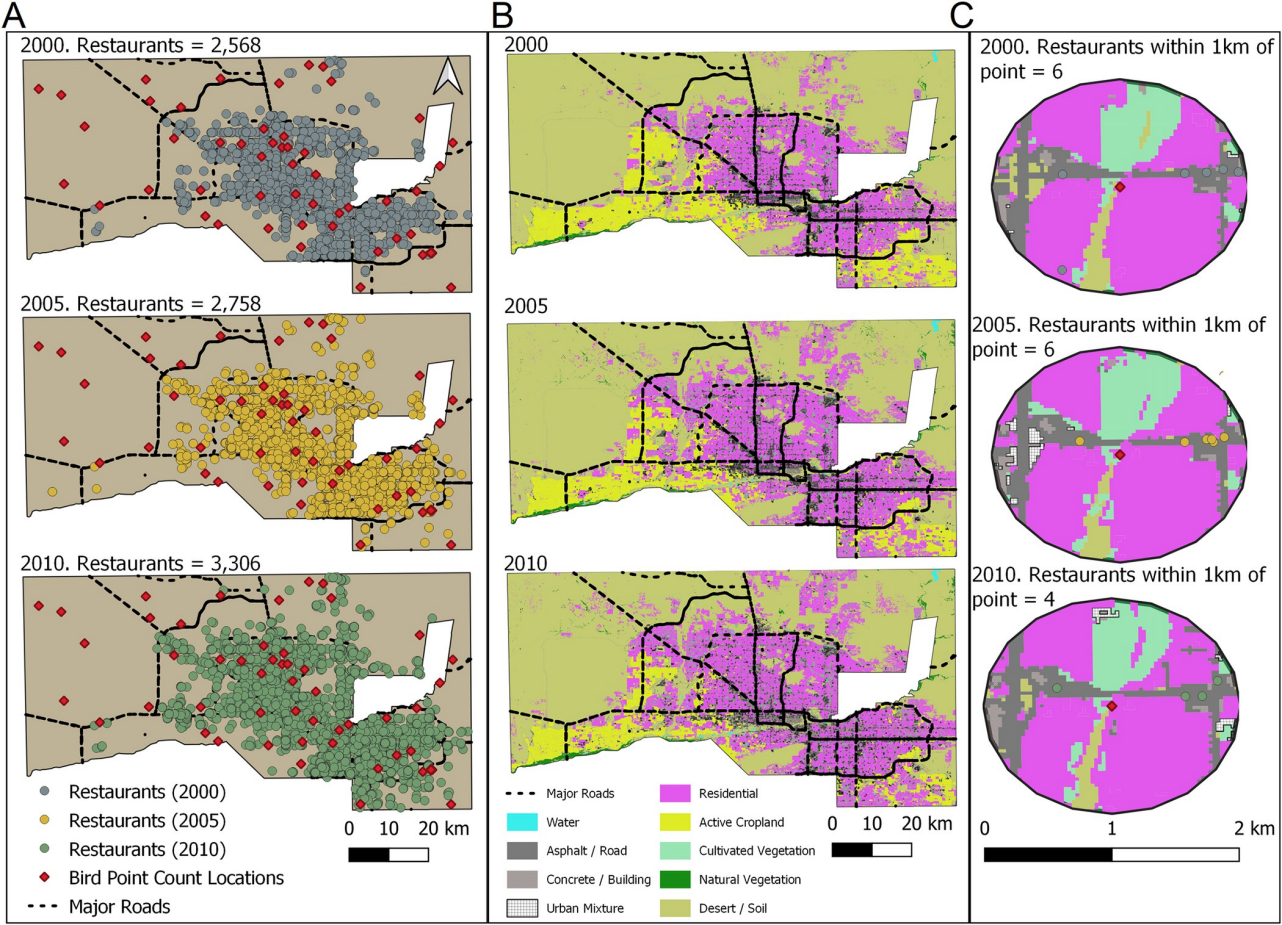
Within the CAP LTER study area in Phoenix, Arizona, the number of restaurants and distribution of restaurants are shown (A) along with the land-use land-cover for 2000, 2005, and 2010 (B). Additionally, a sample point count is shown to display how we measured the number of restaurants within 1 km of each point count for 2000, 2005, and 2010 as well as the land-use within 1 km of the point count for each time period (C).

We used business records from the Maricopa Association of Governments to measure the number of businesses and restaurants within the study area for 2000, 2005, and 2010. We used the North American Industry Classification System to identify which businesses were either full-service restaurants, limited-service restaurants, or other businesses which directly served a form of prepared food to consumers, excluding “bars and lounges” and “drinking places” [55]. We refer to the previously stated collection of businesses as restaurants. To estimate which restaurants birds may be accessing, we plotted all point count locations using QGIS (version 3.8, Fig 1) and created a circular buffer with a 1 km radius around each point since 1 km captures the home range / foraging range of most of our focal species [56, 57] (S1 Table). Within each buffer, we measured the number of restaurants for the years 2000, 2005, and 2010. In our models, we also included the total number of businesses within each buffer because businesses tend to be clustered in more developed areas and so the number of businesses can be used as a general proxy for urbanization [58, 59]. The number of total businesses and restaurants within each buffer were correlated, but both were included in the model since the correlation was less than 0.7 (Spearman’s correlation = 0.59 [60]).

### Controlling for land-use and other urbanization factors

In addition to the number of restaurants, we investigated several other factors, including land-use land-cover (LULC) within the 1 km buffer around each point count. By controlling for LULC, we can isolate the potential impacts of restaurants to ensure that species are not responding to factors associated with restaurant abundance (i.e., if restaurants tend to be in areas with more urban cover and less natural vegetation). The LULC classifications came from CAP LTER and were classified using imagery from 2000, 2005, and 2010 [50], at a 30-meter resolution. LULC was classified as follows: asphalt, active cropland, inactive cropland, water, cultivated vegetation, natural vegetation, concrete/building, residential, residential with white roof, urban mixture, and desert/soil (see sustainability.asu.edu/caplter/data/ for access to data and metadata). We used Spearman’s correlations to test relationships between land-use types and combined categories with a correlation over 0.7 [60]. As a result, we combined active and inactive cropland into “cropland”, residential and residential with white roof into “residential” and asphalt, concrete building, and urban mixture into “highly developed”. Using QGIS (version 3.8), we calculated the percent of each LULC category within 1 km of each point count (Fig 1). We measured the distance each point count was from water (in meters) because the distribution of some species, e.g., those in the *Columbidae* family, including rock pigeons (*Columba livia*) and mourning doves (*Zenaida macroura*), are closely associated with water [61]. However, due to the correlation between distance from water and the amount of water around a point count, this variable was not included. We also ran correlations between LULC, restaurant, and business abundances to ensure that LULC was not directly correlated with restaurant and business distribution (no correlation over 0.7 was seen [60]).

### Richness, total abundance, presence, and species abundance

Richness represents the number of unique species observed at a site during a given season-year combination (e.g., winter 2000 or spring 2005). Since accurately calculating richness and abundance is difficult for rare species, a species needed to be observed at least twice to be included in the analysis [62]. Total bird abundance was measured by adding the maximum number of birds observed at a site within a year for a given season. Species presence was measured as a binary variable (0 or 1) and indicated whether a species was observed at least twice during a given season-year combination. Finally, the abundance for a specific species at each site was measured as the average number of that species seen during all visits for a specific year and season combination (e.g., Spring 2000, Winter 2005).

### Analysis

#### H1: Species abundance and presence in relation to restaurant abundance

We ran a Spearman’s correlation in R [63] between both individual species abundance and species presence/absence and restaurant abundance to identify potential species that might utilize or avoid restaurants. For the correlation analysis, we selected the 20 most abundant species, which constituted 80% of all observed species, and four additional species (rosy-faced lovebird, *Agapornis roceicollis*, Eurasian collared dove, *Streptopelia decaocto*, mallard *Anas platyrhynchos*, and brown-headed cowbird, *Molothrus ater*, S1 Table) that were of interest because they are either non-native or associated with human-influenced landscapes [64, 65]. We limited our species-specific analyses to these species as the larger sample size allowed for more accurate model predictions, and the high abundance or non-native status of the species selected are most likely to alter overall avian communities [66, 67]. For all twenty-four of our focal species, we constructed full global models to compare species presence and species abundance with restaurant abundance, total business abundance, year, and the other described land cover variables (see above) using a generalized linear mixed model (GLMM, [68, 69]). Since our predictive variables were on different scales (i.e., counts versus percent), we scaled all predictive variables using z-scores [70]. To assess the relative importance of each of our measured variables, we used the dredge function in MuMin to run all permutations of our global models and then reported results from the top model using AIC selection [71, 72]. Once top models were confirmed, we used conditional model averaging to calculate beta estimates for each variable within the top models and also calculated the relative importance for each variable in our top models [73]. For each species, we ran a total of four models. We ran two models, one for winter and one for spring, for species presence, and two models, one for winter and one for spring, for species abundance for a total of four models. We ran separate models for each season due to the migratory nature of birds that results in two distinct communities in the spring and winter in our study site [45]. Previous research confirms that stronger predictive results are achieved through individual model creation as opposed to using seasonality as a fixed effect [74]. In our models, presence and species abundance were used as the dependent variables, with presence following a binomial distribution and abundance following a Poisson distribution. In both models, site was our random effect, and all other variables were fixed effects.

Additionally, we investigated whether total species richness and total species abundance differed by site using similar methods but for all species observed more than a single time during a year. In our species richness model, we calculated richness as the total number of species present at a site during a given time and used this as the dependent variable. For total species abundance, we calculated the sum of all individual species abundances as our dependent variable. As in the individual species models, we assessed the relative importance of each of our measured variables by running all permutations of our global models and then reported results from the top model using AIC selection [71, 72]. Once top models were confirmed, we used conditional model averaging to calculate beta estimates for each variable and the relative importance of each variable in the top models [71, 73].

Model 1:

$${Species\;Presence}_{ij}\;or\;{Richness}_{ij}∼{Restaurant\;Abundance}_{ij}+{Business\;Abundance}_{ij}+{Land\;Cover\;Classifications}_{ij}\;{+Year}_{j}+(1|{Site\;ID}_{i})$$

Model 2:

$${Species\;Abundance}_{ij}\;or\;T{otal\;Abundance}_{ij}∼{Restaurant\;Abundance}_{ij}+{Business\;Abundance}_{ij}+{Land\;Cover\;Classifications}_{ij}+{Year}_{j}+(1|{Site\;ID}_{i})$$

where *i* is a point count and *j* is the year the variable was measured.

Since the interpretation of relative importance can vary greatly and be unrelated to predictive power in ecological models, we also created null models to establish the relative importance of variables if they have no predictive power [71, 75, 76]. For the null models of winter and spring individual species presence, we assigned a value of present (1) or absent (0) for each site and year combination drawn from a binomial distribution based on a single draw with a probability equal to the average presence for all focal species across all sites (0.52 for the winter and 0.46 for the spring). We then ran our species presence model using the randomly assigned values as the dependent variable and followed the methods described above to calculate relative importance of each variable. We refer to the output that resulted from running our model on randomized data as the null. We followed this procedure one hundred times and averaged the highest relative importance value from each iteration. We used this average as a threshold and denoted that relative importance values less than those from the null likely indicate the variable is of limited predictive power. We followed a similar method to create thresholds for individual species abundance in the winter and spring, total abundance in the winter and spring, and species richness in the winter and spring. For individual species abundance in the winter and spring, randomly assigned values were drawn from a Poisson distribution with a lambda of 1.97 for the winter and a lambda of 3.38 for the spring. A Poisson distribution was also used to generate random values for total abundance in the winter (lambda = 92.1) and spring (lambda = 201). Lastly, values for richness were drawn from a distribution with a lambda of 21 for the winter and 24.2 for the spring.

#### H2: Community dissimilarity and restaurant abundance

We first calculated community dissimilarity at each site over time for the spring and winter season to test whether restaurants act as predictable food sources that, in turn, stabilize communities over time. Based on all point counts across years for species observed at least twice, communities consisted of a potential 163 species during the winter season and 141 species during the spring. For each site at each time period, community composition was measured as the presence/absence of all potential species during the corresponding season. We then compared the dissimilarity at a site over time using a multiple response permutation procedure (MRPP) using the vegan package in R [77, 78]. Once we had the dissimilarity scores for all sites, we used a generalized linear mixed-model to measure the relationship between the site dissimilarity scores (dependent variable, gaussian distribution) and the variables of interest (independent variables, i.e., LULC, restaurant abundance, business abundance). As in previous models, independent variables were scaled using z-scores [70].

$${Dissimilarity}_{ij}∼{Restaurant\;Abundance}_{ij}+{Business\;Abundance}_{ij}+{Land\;Cover\;Classifications}_{ij\;}+{Year}_{j}+(1|{Site\;ID}_{i})$$

where *i* represents a point count and *j* is the year the variable was measured.

Once the global model was built, we used the dredge function in MuMin to run all permutations of this model and then reported results from the top model using AIC selection [71, 72]. Once top models were confirmed, we used conditional model averaging to create beta estimates for each variable within the top models and associated p-values for those variables [71, 73].

#### H3: Community composition and restaurant abundance

To investigate whether restaurant abundance influences overall avian community composition, presence (0 or 1) for all species for the spring and winter season was used to conduct non-metric multidimensional scaling (NMDS). We used the vegan package in R [78] to conduct the NMDS. Fit for NMDS was tested using stress plots. Additionally, we clustered sites by the number of restaurants within 1 km of the point count based on the overall distribution of restaurants in the study site. We used these clusters to investigate if the number of restaurants impacts community dynamics. Sites with no restaurants during a given time period were classified as “none” (total n = 109, n for year 2000 = 37, n for year 2005 = 35, n for year 2010 = 49), sites with two or fewer restaurants were classified as “low” as both the mean and median number of restaurants across sites was two (n = 19), and sites with more than two restaurants were classified as “high” (n = 37). We then used PERMANOVA to test if these groups were significantly different in their clustering and used the betadisper function to check for differences in dispersion [71, 78]. We ran all analyses using R version 3.6.1 [79].

## Results

### Restaurants and businesses

We documented an increase in the number of businesses within the Phoenix metropolitan area and the number of restaurants from 2000 to 2010 (Table 1). Despite increases in the number of total businesses, not all businesses and restaurants persisted from 2000 until 2010 as some businesses and restaurants permanently closed with ~55% of businesses open in 2000 remaining open in 2010 in the same location. As a result, the average number of businesses within 1 km of point counts showed an increasing trend, but there was no significant difference between the number of businesses within 1 km of point counts in 2000, 2005, and 2010 (ANOVA, p = 0.11, df = 287, F = 1.21). The number of restaurants did not increase at the same rate as general businesses and within 1 km of point counts the number of restaurants between years did not significantly differ (ANOVA, p = 0.59, df = 287, F = 0.09; Table 1).

**Table 1:** The number of registered businesses and restaurants for each year is presented along with the average number of businesses and restaurants within the 1 km buffer of each point count. Minimum and maximum of # of businesses and restaurants within 1 km buffer are presented in parenthesis (min | max).

Year	# Businesses	Average # Businesses within 1 km	# Restaurants	Average # Restaurants within 1 km
2000	33,638	25.12 (0 | 185)	2,568	1.83 (0 | 28)
2005	37,566	27.17 (0 |193)	2,758	2.01 (0 | 33)
2010	46,924	31.74 (0 | 225)	3,306	2.05 (0 | 26)

### H1: Species abundance and presence in relation to restaurant abundance

A total of 19,559 observations were recorded during the three sampling years of the study across the 57-point count locations. These observations accounted for 41,429 individual birds comprising 187 species.

### Species specific models

**Fig 2:**
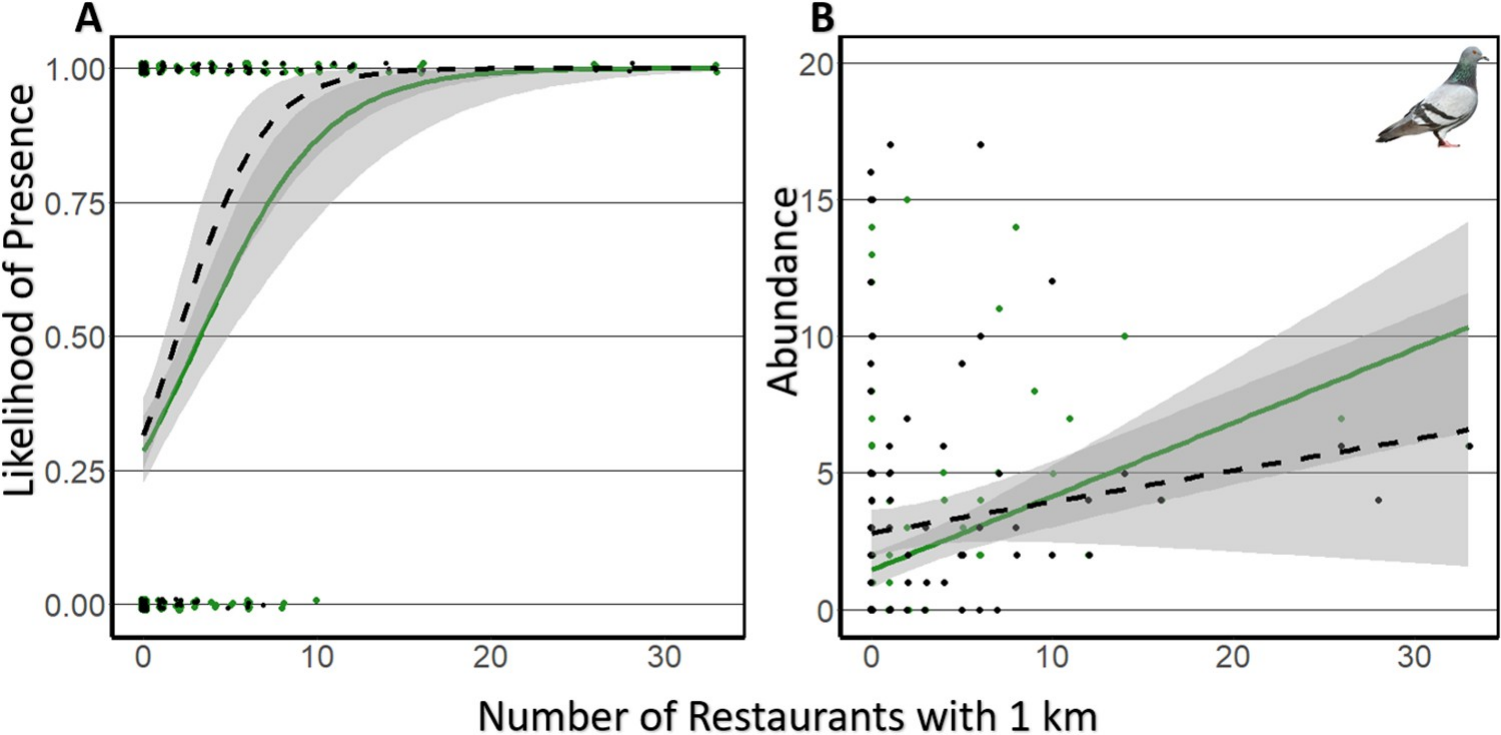
Significant correlations between restaurant abundance and rock pigeon, the most common species associated with restaurants. Each point represents the species presence (A) or abundance (B) for spring (green) or winter (black). Trendlines for spring (green) and winter (black dashed) are shown as well as their associated 95% confidence intervals in gray. Note that points for presence (A) are jittered to better display the information. A total of 128 site and year combinations are included for the spring and 161 site and year combinations are included for the winter. For the spring, 29% of all sites are occupied representing a total abundance of 304 individuals. For the winter, 51% of all sites are occupied accounting for 479 individuals.

For individual species models, for nine of the twenty-four species investigated, the number of restaurants within 1 km of the point count was a potentially meaningful predictor of either species abundance or species presence when accounting for land-cover and other metrics of urbanization (Fig 2, Table 2, S2–S5 Tables). For individual species abundance, the null model predicted an average maximum variable importance of 0.55 for the winter and 0.63 for the spring. During the winter, the abundance of mourning doves (*Zenaida macroura*), rock pigeons (*Columba* livia), and great-tailed grackles (*Quiscalus mexicanus*) was positively associated with restaurant count whereas the abundance of house sparrows (*Passer domesticus*), house finches *(Haemorhous mexicanus*), and European starlings (*Sturnus vulgaris*) was negatively associated with restaurant count (S2 Table). For individual spring abundance models, the abundance of three species, mourning doves, rock pigeons, and Inca doves (*Columbina inca*), was positively associated with restaurant abundance whereas the abundance of red-winged blackbirds (*Aeglaius phoeniceus*) was negatively associated with restaurant abundance (S3 Table). For the winter presence models, only a single species, red-winged blackbirds, were associated with restaurant abundance. For red-winged blackbirds, as restaurant counts increased, presence decreased (S4 Table). For four of the spring species presence models, the relative importance of restaurants in contributing to top models was greater than the maximum averaged null relative importance of 0.70. Based on our models, the presence of both mourning doves and rock pigeons is predicted to increase with restaurant abundance whereas the presence of house finches and brown-headed cowbirds (*Molothrus ater)* is predicted to decrease with increasing restaurant abundance (S5 Table).

**Table 2:** The beta estimates for the influence of restaurants on species abundance and species presence for winter and spring. The maximum relative importance value based on null model predictions is presented next to the abundance and presence columns. Beta estimates are from averaged models where the relative importance exceeds the null (see S2–S5 Tables, for full model details). The relative importance value for restaurants is presented next to the beta estimates. Dashes indicate the relative importance value for restaurants below the null model values.

	Abundance		Presence	
Species	Winter (0.55)	Spring (0.63)	Winter (0.66)	Spring (0.70)
Mourning Dove (*Zenaida macroura)*	0.48 (1)	0.22 (1)	-	0.07 (0.87)
Great-tailed Grackle *(Quiscalus mexicanus)*	0.11 (1)	-	-	-
House Sparrow *(Passer domesticus)*	-0.10 (1)	-	-	-
House Finch (*Haemorhous mexicanus*)	-0.14 (1)	-	-	-0.90 (0.90)
European Starling *(Sturnus vulgaris)*	-0.10 (1)	-	-	-
Rock Pigeon (*Columba livia)*	0.34 (0.92)	0.31 (1)	-	0.91 (0.71)
Red-Winged Blackbird (*Agelaius phoeniceus*)	-	-1.56 (1)	-0.75 (1)	-
Inca Dove *(Columbina inca)*	-	0.43 (1)	-	-
Brown-headed Cowbird *(Molothrus ater)*	-	-	-	-0.94 (1)

For individual species abundance and presence models in both the winter and spring, land-cover and the number of businesses were also given relative importance values higher than the corresponding null and thus the number of restaurants was not the only indictor of presence or abundance (see discussion and S2–S5 Tables).

### Site specific models

The average richness observed during the winter period was 21 species with a minimum of eight and a maximum of 55 species observed. For winter richness models, nine models fell within a delta AIC (DAIC) of 2 with the number of businesses, the amount of highly developed land, number of restaurants, amount of natural vegetation, water, residential land, soil/desert, and cropland all appearing in top models (S6 Table). The conditional averaged model, which weighted the conditional importance of each variable [73], contained six variables with a relative importance above the estimated null relative importance of 0.65. These include the number of businesses, the amount of highly developed land, the number of restaurants, the year, the amount of natural vegetation, and the amount of cultivated vegetation. Increasing the number of businesses, the amount of highly developed land, the year, the amount of natural vegetation, and the amount of cultivated vegetation are all associated with increasing species richness during the winter. In contrast, the number of restaurants negatively influenced species richness (S6 Table).

The average richness observed during the spring period was 24 species with a minimum of three species and a maximum of 65 species. For the spring richness models, two models were within 2 DAIC of the top model. The first model included the number of businesses, cropland, cultivated vegetation, highly developed land, natural vegetation, residential land, soil/desert, water, and the year. The second model was the global model–it included all previous variables as well as the number of restaurants (S7 Table). However, although the number of restaurants appeared within the top two models, its relative importance of 0.24 fell below the average maximum relative importance calculated based on randomized data (0.71) and thus the number of restaurants may not meaningfully predict species richness during the spring.

Nine models were within 2 DAIC of the top models for winter abundance. The global model was included in the top model, indicating that each measured variable helps explain some variations in the species abundance at sites during the winter. Additionally, the relative importance of all variables fell above the relative importance calculated based on randomized abundance values (0.68) and thus each variable is likely associated with species abundance during the winter (S8 Table). The number of businesses and the amount of residential land positively correlated with species abundance, whereas increasing the number of developed land, restaurants, soil/desert, cropland, cultivated vegetation, natural vegetation, the year, and the amount of water was negatively associated with species abundance. Despite the high relative importance of all these variables, the effect size of the year was by far the highest indicating that while other variables may be meaningful, overall trends in species abundance are driven primarily by year. This matches previous work highlighting avian population declines in the study area [74].

For spring abundance, the global model was the best fitting model and was eight DAIC away from the second-best model. Residential land, soil and desert, cropland, and highly developed land had the strongest significant positive correlation with species abundance during the spring. The only negatively related variable was the year. As the year increased, the abundance of species decreased (S9 Table).

### H2: Community dissimilarity and restaurant abundance

For analyses of community turnover in the winter season, 17 models were within 2 DAIC ([71], S10 Table) of the top model. The averaged maximum relative importance value calculated from our randomized trials was 0.38 and six of the variables from our top models had a relative importance greater than 0.38. The number of restaurants was included in 15 of the top models and had the highest relative importance of all variables (0.92) and had a negative beta estimate suggesting that the bird communities that associated with the restaurants are more like each other than communities not associated with restaurants (-0.05, Tables 3 and S10). The amount of highly developed land, residential land, number of businesses, and year also decreased community dissimilarity and therefore may contribute to biotic homogenization. The contribution of all these variables is likely not equal however, as the amount of developed land had a much larger effect than the number of restaurants or businesses. This may indicate that while the number of restaurants is frequently associated with biotic homogenization (high relative importance), the overall contribution of restaurants to biotic homogenization may be lesser than other elements of urbanization. Unsurprisingly, conditional beta estimates show that natural vegetation is likely to increase community dissimilarity as natural areas are likely less uniform than urban ones (Table 3).

**Table 3:** Results from the model averaging (DAIC <2) of the models investigating the relationship between community dissimilarity and variables of interest during the winter. Model averaged conditional beta estimates as well as relative importance and the number of top models the variable was included in. The variables are listed by relative importance.

Variable	Estimate	Relative Importance	Number of Models Included
Restaurants	-0.05	0.92	15
Natural Vegetation	0.51	0.81	14
Highly Developed	-0.43	0.48	8
Residential	-0.17	0.47	8
Businesses	-0.01	0.47	8
Year	-1.12	0.43	7
Cultivated Vegetation	0.05	0.22	3
Cropland	-0.37	0.19	3
Soil / Desert	-0.38	0.19	3
Water	0.18	0.12	2

For the spring season, there were five models within 2 DAIC [60] of the top model. Although restaurants appeared in two of the top models, the relative importance of restaurants was less than the relative importance calculated from our randomized trials (0.55) indicating that the number of restaurants is not meaningful in predicting biotic homogenization in the spring. However, the amount of natural vegetation, water, developed area, and soil/desert all had relative importance greater than 0.55 (Table 4, S11 Table). Bird communities observed in areas with high amounts of natural vegetation, soil/desert, and water were more dissimilar to each other over time, while bird communities observed in highly developed areas were more similar to each other over time (Table 4).

**Table 4:** Results from the model averaging (DAIC <2) of the models investigating the relationship between community dissimilarity and variables of interest during the spring. Model averaged conditional beta estimates as well as relative importance and the number of top models the variable was included in. The variables are listed by relative importance.

Variable	Estimate	Relative Importance	Number of Models Included
Natural Vegetation	0.29	1.00	5
Water	0.14	1.00	5
Soil / Desert	0.36	0.84	4
Highly Developed	-0.23	0.82	4
Restaurants	-0.09	0.33	2
Cultivated Vegetation	0.17	0.16	1
Cropland	-0.13	0.16	1
Residential	-0.31	0.16	1

### H3: Community composition and restaurant abundance

**Fig 3:**
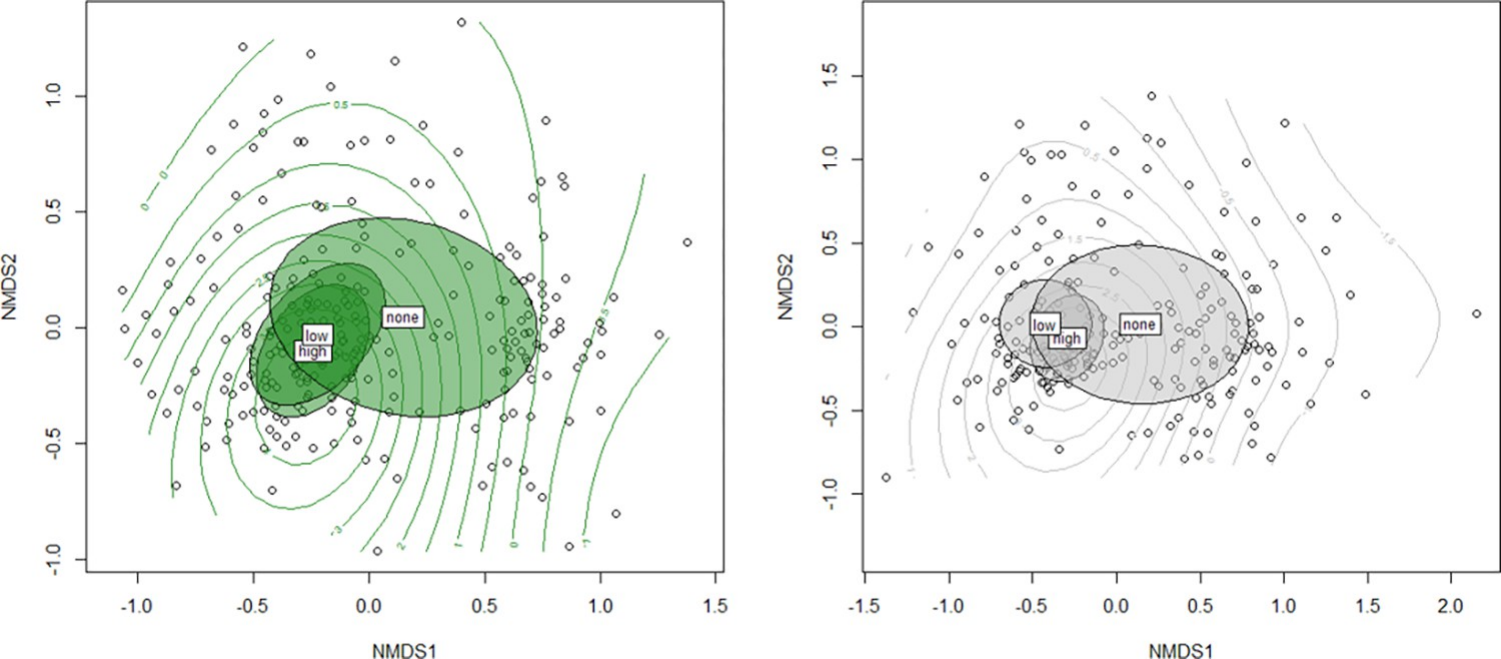
Communities for all sites from 2000, 2005, and 2010 during the spring season are plotted as green points in the left panel where communities from the winter season are plotted on the right. Clusters for “high”, “low”, and no restaurants are shown as ellipses overlapping the sites. Contour lines are used to display the gradient of increasing restaurants.

For the spring season, bird communities did not differ between sites with “low” and “high” restaurant abundance (PERMANOVA, p = 0.71; [78, 80]) but sites with either “low” or “high” restaurant abundance differed from sites with no restaurants (PERMANOVA, p < 0.05, Fig 3; [62]). Additionally, results from the dispersion test [78, 80] show that sites with at least one restaurant have more similar bird communities (i.e., there is less dissimilarity between communities resulting in tighter clusters) than sites with no restaurants (p < 0.05 for the difference between “low”/“high” sites and no restaurant sites). However, there was no difference in dispersion between the “low” and “high” restaurant sites (p = 0.59). A similar pattern was seen for the results of the winter season analysis. Sites with “low” restaurant counts and “high” restaurant counts had similar dispersion (p = 0.22), but both had lower dispersion than sites with no restaurants (p < 0.00 for both “low” and “high” restaurants). Unlike the spring analysis, however, sites with “low” restaurant abundance differed in community composition from sites with “high” restaurant abundance (p < 0.05) during the winter. All sites with restaurants, regardless of the amount, had significantly different bird communities compared with sites with no restaurants (p < 0.06 for both “low” and “high” restaurant sites, Fig 3). Sites with restaurants tended to have fewer species overall and almost all sites with restaurants contained rock pigeons and mourning doves. In contrast, both species were less common at sites without restaurants. Results from the stress plot indicate the NMDS for both the spring and winter seasons accurately represented the data (spring R^2^ = 0.96, winter R^2^ = 0.93 as via Shepard diagram [81]).

## Discussion

As part of rapid urban development, restaurants have become a common component of the landscape and shape urban wildlife communities. We found a relationship between the abundance of restaurants and the abundance and presence of nine avian species providing evidence for the partial confirmation of H1 (the number of restaurants at a location influences species abundance and presence). Four of the nine species showed a positive relationship between species abundance and restaurant abundance. These species are among the most widespread urban species, and they may influence the broader urban avian community through species interactions [18, 82]. Community variability decreased in areas with higher restaurant abundance, highlighting the possibility that, in acting as predictable food sources, restaurants stabilize communities over time, providing evidence for H2 (restaurants act as predictable food sources that stabilize communities). Finally, our data highlights the probability of H3 (restaurant density influences avian community composition) because bird communities at sites with restaurants are more like each other than they are to sites without restaurants. The increased similarity suggests that restaurants influence avian community composition and might serve as a homogenizing force [31]. The resulting homogenization may strongly inform urban conservation practices as many conservation efforts center on preserving existing biodiversity, and thus, increased homogenization may limit conservation efficacy [83]. Further, biotic homogenization may also limit human-wildlife interactions and contribute to the extinction of experiences with wildlife [28, 84, 85].

Although urban areas are highly heterogenous, restaurants are a common feature throughout. In the United States alone, there are over a million registered restaurants and these restaurants cluster in areas of high population densities (i.e., urban areas, [86, 87]) and produce large amounts of food waste [42–44]. Our results provide some support that restaurants may elevate specific species abundance, but overall trends are mixed with total species abundance increasing with restaurants during the spring and decreasing during the winter. The differences observed in the influence of restaurants during the spring and winter may relate to the availability of resources in the system as well as the life history and behavior of bird species. Food resources may be less available in the winter than at other times. If so, restaurants may allow for some bird species to persist in larger numbers and for larger flock sizes outside of the breeding season [46, 88, 89]. In addition, restaurants might act as stable food sources. Stable food sources provided by restaurants may help explain why urban biological communities go through fewer boom and bust cycles than their rural counterparts [90] and why several species, including great-tailed grackles, now overwinter in urban areas instead of migrating to track insect populations [46, 47]. However, while stable food sources may link to the persistence and increase of specific species during the winter, total species abundance may not increase with restaurant abundance during the winter if birds do not need supplemental resources. During the spring, birds often are reproducing and caring for young, which requires additional energy that restaurants might provide [91]. During the winter, birds may be less reliant on urban resources as seen with the negative correlation between both restaurants and developed land-use land cover in the total abundance model. Despite a lack of connection to restaurant abundance, there is a positive correlation between species abundance and businesses during the winter, which highlights potential elements of urbanization, such as warm areas to shelter, that may be more important than food during non-breeding stages of a bird’s life [92].

When examining specific species, the trend for increased species abundance associated with restaurants is most pronounced with mourning doves and rock pigeons and remains positive for both spring and winter. These species were found at nearly every site with at least one restaurant but were rarely found at sites without restaurants. They are also commonly found in cities throughout North America and have been documented utilizing anthropogenic food sources [93, 94]. The potential link between the increase in urban-exploiting species and restaurant abundance also highlights the role restaurants may play in promoting the persistence and spread of non-native species such as the rock pigeon. Anthropogenic food sources have also been highlighted as a route for non-native bird establishment in the case of the monk parakeet (*Myiopsitta monachus*) in Chicago, Illinois, USA [88] and rose-ringed parakeets (*Psittacula krameria*) in London, England [17]. Although rock pigeons have been established in North America since the 17^th^ century [95], their continued persistence and success in urban environments may be in part due to anthropogenic food sources. Urban landscapes often host a wide variety of non-native species because they provide novel resources and a mix of habitat features [12, 66, 67]. Rock pigeons are among the most ubiquitous urban species and can utilize a wide range of anthropogenic food resources. Rock pigeons may also outcompete native species for resources including food and nesting space, and may spread disease to native species [96, 97]. However, restaurants may skew the distribution of urban-exploiting species toward those species which can best utilize the resource. Although both rock pigeons and mourning doves are commonly observed utilizing resources from restaurants (S2 Fig), our research provides empirical evidence that this relationship may occur frequently and at city-wide scales. Additionally, experimental research demonstrated the ability of pigeons and other Columbidae to subsist on diets consisting of foods commonly associated with restaurants (e.g., highly refined foods including white bread and french-fries; [26, 98]). The previous research suggests that Columbidae may be highly adept at using these resources leading to an overall increase in their abundance. Some Columbidae show a strong association with agrarian landscapes, which they may utilize for food [99, 100]. When fields become inactive during the winter, these birds may turn to restaurants as a food source.

Our study shows that sites near restaurants have lower species diversity and the communities at these sites tend to be similar to each other. Thus, restaurants, and stable food sources in general, may contribute to urban homogenization [24, 26]. Predictable food sources from restaurant refuse may play a larger role in dynamic environments where food sources are typically patchy or seasonal. The abundance of mourning doves and rock pigeons shows a stronger positive relationship with restaurant abundance during the winter than in the spring. The seasonal dynamic may be especially strong in the Phoenix metropolitan area and other desert systems, which receive most of their rain in a short period and, as a result, have high temporal variability in the vegetative community. Additionally, restaurants may also contribute to biotic homogenization by indirectly promoting competitive exclusion [82]. The three species positively associated with restaurants comprised 8% of total bird abundance in both the spring and winter seasons. While restaurants may provide food sources for these species, they are likely not the only food source utilized. Columbidae likely still compete with other species for nesting or sheltering locations and additional food sources [101, 102]. Reducing the availability of anthropogenic resources through more regulated waste management protocols, such as closed trash cans and dumpsters, may limit the potentially homogenizing role of restaurants in urban environments. Potential competitive exclusion may also impact human well-being since most human-wildlife interactions occur within urban landscapes, and these interactions may shape individuals’ perceptions toward wildlife in general [103]. Further, individuals’ perceptions of wildlife are related to the characteristics of the species they interact with. For birds, people have more positive attitudes towards colorful species [104, 105]. Since rock pigeons, Inca doves, and mourning doves are primarily gray species, their increase in prevalence could have negative impacts on people and their perception of wildlife. In turn, negative perceptions of wildlife may limit individuals’ future participation or support for wildlife conservation [103].

Our study highlights the potential impact restaurants may have on avian communities, but we cannot definitively conclude that it is the food that restaurants provide, specifically, that influences avian communities. Indeed, restaurants can provide food but also other resources. Particularly, in Phoenix, many restaurants may also act as cooler microclimates [106]. In the spring and summer, restaurants often provide misters around their outdoor seating areas to keep patrons comfortable. These cooler areas may also reduce the impacts of extreme heat, which species could otherwise not tolerate [106]. Further, restaurants may be associated with other factors that alter avian abundance like noise [107, 108] and may indirectly alter other food sources (e.g., attracting insects via outdoor lighting or trash; [109]). With potential impacts on both wildlife and people, ecologists and city planners could further investigate the influence of restaurants on urban wildlife through mechanistic studies [110, 111]. Since our study cannot identify restaurants as the driver of the documented patterns, we suggest future research take on an experimental and mechanistic approach to fully understand the influence of restaurants on avian communities. Lastly, studies that investigate wildlife community change prior to the opening of a new restaurant or after the closing of a restaurant can highlight temporal dynamics in community change and provide planners with a stronger understanding of how the inclusion of a restaurant in urban design may alter wildlife populations.

## Supporting information

S1 Fig The Phoenix Metropolitan area’s location is highlighted in the map of North America in the top right. The CAP LTER study area the associated land cover categories from 2010 within the study area are shown.
(DOCX)

S2 Fig Photographs by Jeffrey Brown show rock pigeons and great-tailed grackles competing for food outside a restaurant in Phoenix, AZ. (DOCX)

S1 Table Total abundance for the 24 species investigated in the study. Total abundance is the total number of individuals observed across the study. Average abundance is the average value across all years for a given species per site per year. Presence is the proportion of sites the species was present at averaged across the three time periods. Species are ordered by total abundance.
(DOCX)

S2 Table Standardized conditional beta estimates from model averaging for individual species abundance during the winter. The relative importance of each variable is displayed next to the standardized beta estimate in parentheses. The total number of models with 2 DAIC of the top model are displayed before the species name. The maximum relative importance value calculated based on the random null model (see methods) was 0.55. All variables with a relative importance value above 0.55 are bolded for emphasis. Asterisks indicate the standardized beta estimate’s 95% confidence intervals do not overlap with zero.
(DOCX)

S3 Table Conditional beta estimates from model averaging for individual species abundance during the spring. The relative importance of each variable is displayed next to the standardized beta estimate in parentheses. The total number of models with 2 DAIC of the top model are displayed before the species name. The maximum relative importance value calculated based on the random null model (see methods) was 0.63. All variables with a relative importance value above 0.63 are bolded for emphasis. Asterisks indicate the standardized beta estimate’s 95% confidence intervals do not overlap with zero.
(DOCX)

S4 Table Conditional beta estimates from model averaging for individual species presence during the winter. The relative importance of each variable is displayed next to the standardized beta estimate in parentheses. The total number of models with 2 DAIC of the top model are displayed before the species name. The maximum relative importance value calculated based on the random null model (see methods) was 0.66. All variables with a relative importance value above 0.66 are bolded for emphasis. Asterisks indicate the standardized beta estimate’s 95% confidence intervals do not overlap with zero.
(DOCX)

S5 Table Conditional beta estimates from model averaging for individual species presence during the spring. The relative importance of each variable is displayed next to the standardized beta estimate in parentheses. The total number of models with 2 DAIC of the top model are displayed before the species name. The maximum relative importance value calculated based on the random null model (see methods) was 0.70. All variables with a relative importance value above 0.70 are bolded for emphasis. Asterisks indicate the standardized beta estimate’s 95% confidence intervals do not overlap with zero.
(DOCX)

S6 Table Relative importance of variables within the top models (DAIC <2) for estimates of winter species richness by site. Below, relative importance are the standardized conditional beta estimates with upper and lower 95% confidence intervals for each variable based on the variable’s relative importance across all top models. The top nine models are also displayed below with + indicating the variable is included in the model. Our randomized null model contained variables with estimated relative importance of 0.65, thus variables with a relative importance above 0.65 likely have meaningful predictive power. The variables are listed by relative importance.
(DOCX)

S7 Table Relative importance of variables within the top models (DAIC <2) for estimates of spring species richness by site. Below, relative importance are the standardized conditional beta estimates with upper and lower 95% confidence intervals for each variable. The top two models are displayed below with + indicating the variable is included in the model. Variables are listed by relative importance. Our randomized null model contained variables with estimated relative importance of 0.71, thus variables with a relative importance above 0.71 likely have meaningful predictive power.
(DOCX)

S8 Table Relative importance of variables within the top models (DAIC <2) for estimates of winter species abundance by site. Below, relative importance are the standardized conditional beta estimates for each variable based on the variable’s relative importance across all top models. 95% confidence intervals are shown for each beta estimate in parathesis. The top nine models are displayed below with + indicating the variable is included in the model. Our randomized null model contained variables with estimated relative importance of 0.68, thus variables with a relative importance above 0.68 likely have meaningful predictive power. Variables are listed by relative importance.
(DOCX)

S9 Table Relative importance of variables within the top models (DAIC <2) for estimates of spring species abundance by site. No models were within 2 DAIC of the top model. Below, relative importance are the standardized conditional beta estimates for each variable based on the variable’s relative importance across all top models. 95% confidence intervals are shown for each beta estimate in parathesis. The top model is displayed below with + indicating the variable is included in the model. Our randomized null model contained variables with estimated relative importance of 0.73, thus variables with a relative importance above 0.73 likely have meaningful predictive power. The variables are listed alphabetically.
(DOCX)

S10 Table Relative importance of variables within the top models (DAIC <2, 17 models) for assessing community dissimilarity during the winter season. Below, relative importance are the standardized conditional beta estimates for each variable based on the variable’s relative importance across all top models. 95% confidence intervals are shown for each beta estimate in parathesis. The top nine models are displayed below with + indicating the variable is included in the model. Our randomized null model contained variables with estimated relative importance of 0.38, thus variables with a relative importance above 0.38 likely have meaningful predictive power. The variables are listed by relative importance.
(DOCX)

S11 Table Relative importance of variables within the top models (DAIC <2, 5 models) for assessing community dissimilarity during the spring season. Below relative importance are the standardized conditional beta estimates for each variable based on the variable’s relative importance across all top models. 95% confidence intervals are shown for each beta estimate in parathesis. The top nine models are displayed below with + indicating the variable is included in the model. Our randomized null model contained variables with estimated relative importance of 0.55, thus variables with a relative importance above 0.55 likely have meaningful predictive power. The variables are listed by relative importance.
(DOCX)
