# Supplementary material for: No fry zones: How restaurant distribution and abundance influence avian communities in the Phoenix, AZ metropolitan area
Source: PLoS One. 2022 Oct 19;17(10):e0269334. doi: 10.1371/journal.pone.0269334 (PMC9581420; doi:10.1371/journal.pone.0269334)

Supplemental Materials – No Fry Zones

Supplemental Figure 1: The Phoenix Metropolitan area’s location is highlighted in the map of North America in the top right. The CAP LTER study area the associated land cover categories from 2010 within the study area are shown.
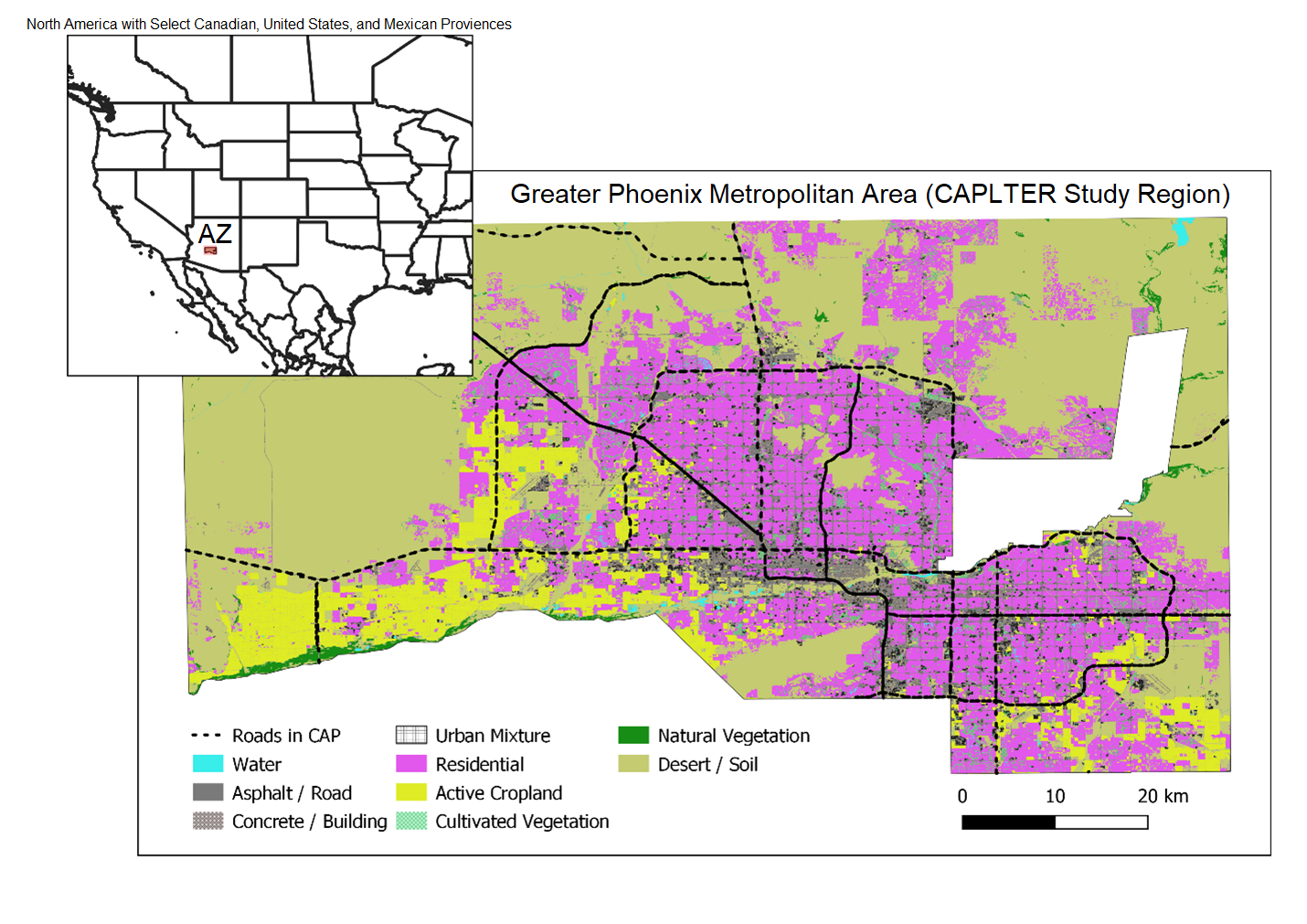

Supplement: S1 Fig — The CAP LTER study area the associated land cover categories from 2010 within the study area are shown. (DOCX) [file pone.0269334.s001.docx]
