# Supplementary material for: No fry zones: How restaurant distribution and abundance influence avian communities in the Phoenix, AZ metropolitan area
Source: PLoS One. 2022 Oct 19;17(10):e0269334. doi: 10.1371/journal.pone.0269334 (PMC9581420; doi:10.1371/journal.pone.0269334)
Supplement: S2 Fig — (DOCX) [file pone.0269334.s002.docx]

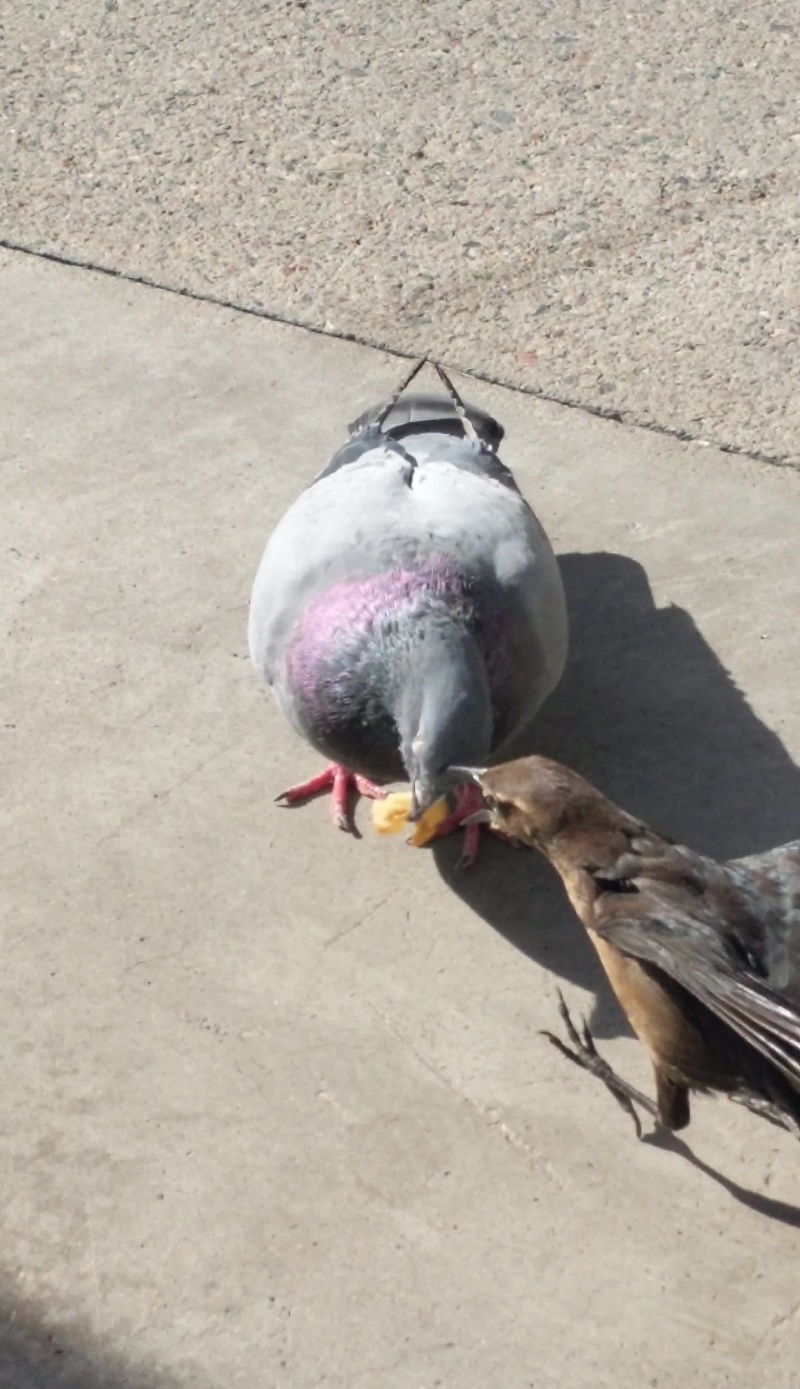

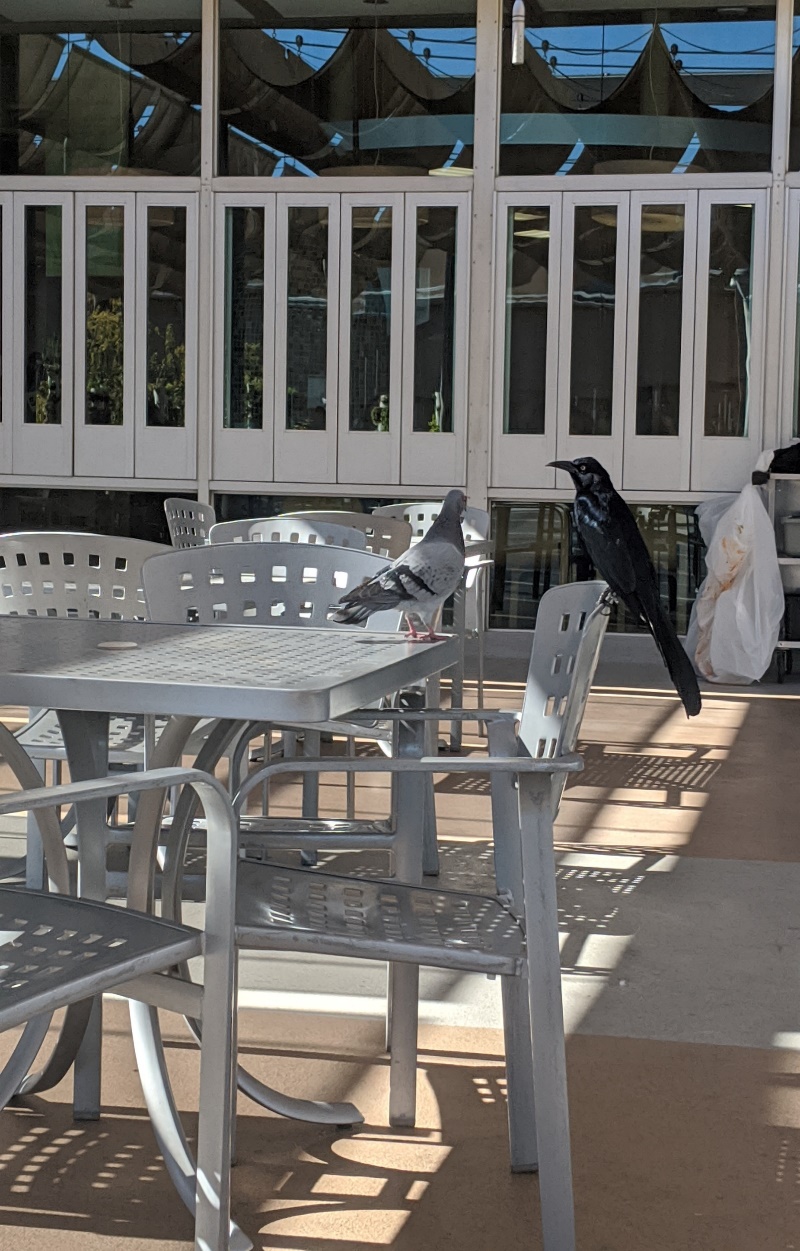
Supplemental Figure 2: Photographs by Jeffrey Brown show rock pigeons and great-tailed grackles competing for food outside a restaurant in Phoenix, AZ.
