# Supplementary material for: No fry zones: How restaurant distribution and abundance influence avian communities in the Phoenix, AZ metropolitan area
Source: PLoS One. 2022 Oct 19;17(10):e0269334. doi: 10.1371/journal.pone.0269334 (PMC9581420; doi:10.1371/journal.pone.0269334)
Supplement: S1 Table — Total abundance is the total number of individuals observed across the study. Average abundance is the average value across all years for a given species per site per year. Presence is the proportion of sites the species was present at averaged across the three time periods. Species are ordered by total abundance. (DOCX) [file pone.0269334.s003.docx]

Supplemental Table 1: Total abundance for the 24 species investigated in the study. Total abundance is the total number of individuals observed across the study. Average abundance is the average value across all years for a given species per site per year. Presence is the proportion of sites the species was present at averaged across the three time periods. Species are ordered by total abundance.

| Species | Total Abundance | Average Abundance (Spring) | Presence (Spring) | Average Abundance (Winter) | Presence (Winter) |
| --- | --- | --- | --- | --- | --- |
| Mourning Dove (*Zenaida macroura)* | 2436 | 5.91 | 0.96 | 12.47 | 0.97 |
| Great-tailed Grackle *(Quiscalus mexicanus)* | 1719 | 3.25 | 0.65 | 12.10 | 0.70 |
| House Sparrow *(Passer domesticus)* | 1672 | 3.91 | 0.64 | 6.71 | 0.67 |
| House Finch (*Haemorhous mexicanus*) | 1304 | 3.13 | 0.71 | 9.48 | 0.99 |
| European Starling *(Sturnus vulgaris)* | 987 | 2.47 | 0.56 | 5.24 | 0.71 |
| White-winged Dove (*Zenaida asiatic)* | 838 | 10.64 | 0.75 | 0.67 | 0.28 |
| Verdin *(Auriparus flaviceps)* | 785 | 0.79 | 0.60 | 1.25 | 0.87 |
| Rock Pigeon (*Columba livia)* | 783 | 2.38 | 0.38 |  | 0.51 |
| Gila Woodpecker *(Melanerpes uropygialis)* | 733 | 1.26 | 0.61 | 1.06 | 0.77 |
| Northern Mockingbird *(Mimus polyglottos)* | 625 | 0.78 | 0.55 | 0.91 | 0.73 |
| Cactus Wren *(Campylorhynchus brunneicapillus)* | 600 | 0.62 | 0.42 | 0.71 | 0.56 |
| Gambel’s Quail *(Callipepla gambelii)* | 538 | 0.84 | 0.32 | 1.45 | 0.41 |
| Anna’s Hummingbird (*Calypte anna)* | 493 | 0.59 | 0.45 | 0.95 | 0.72 |
| Curve-billed Thrasher *(Toxostoma curvirostre)* | 478 | 0.57 | 0.48 | 0.79 | 0.64 |
| Abert’s Towhee (*Pipilo aberti)* | 345 | 0.58 | 0.28 | 0.84 | 0.53 |
| Red-winged Blackbird (*Agelaius phoeniceus*) | 333 | 4.00 | 0.23 | 10.50 | 0.31 |
| White-crowned Sparrow (*Zonotrichia leucophrys*) | 265 | NA | NA | 2.35 | 0.54 |
| Black-throated Sparrow (*Amphispiza bilineata)* | 250 | 0.45 | 0.22 | 1.14 | 0.33 |
| Inca Dove *(Columbina inca)* | 199 | 0.64 | 0.30 | 0.60 | 0.23 |
| Killdeer *(Charadrius vociferous)* | 187 | 0.52 | 0.27 | 1.11 | 0.31 |
| Brown-headed Cowbird *(**Molothrus ater)* | 180 | 1.03 | 0.31 | 1.34 | 0.29 |
| Mallard *(Anas platyrhynchos)* | 140 | 0.51 | 0.13 | 2.06 | 0.23 |
| Eurasian Collared-Dove *(Streptopelia decaocto)* | 116 | 2.47 | 0.11 | 0.30 | 0.16 |
| Rosy-faced Lovebird *(Agapornis roseicollis)* | 21 | 2.34 | 0.05 | 0.01 | 0.04 |
