# Supplementary material for: No fry zones: How restaurant distribution and abundance influence avian communities in the Phoenix, AZ metropolitan area
Source: PLoS One. 2022 Oct 19;17(10):e0269334. doi: 10.1371/journal.pone.0269334 (PMC9581420; doi:10.1371/journal.pone.0269334)
Supplement: S3 Table — The relative importance of each variable is displayed next to the standardized beta estimate in parentheses. The total number of models with 2 DAIC of the top model are displayed before the species name. The maximum relative importance value calculated based on the random null model (see methods) was 0.63. All variables with a relative importance value above 0.63 are bolded for emphasis. Asterisks indicate the standardized beta estimate’s 95% confidence intervals do not overlap with zero. (DOCX) [file pone.0269334.s005.docx]

| Variable | *Mourning Dove (12)* | *Rock Pigeon (1)* | *Red-winged Blackbird (11)* | *Inca Dove (5)* |
| --- | --- | --- | --- | --- |
| Restaurant Count | **0.22 (1)*** | **0.31 (1)*** | **-1.56 (1)*** | **0.43 (1)*** |
| Business Count | 0.11 (0.58) | **-0.56 (1)*** | **-1.57 (1)*** | -0.52 (0.33) |
| Year | -0.05 (0.38) | **-0.56 (1)*** | **0.37 (1)*** | **-0.19 (0.68)*** |
| Cultivated Vegetation | **0.24 (1)** | **1.41 (1)*** | **0.25(1)** |  |
| Cropland | 1.38 (0.49) | **4.33 (1)*** | 1.05 (0.61) |  |
| Highly Developed | 0.78 (0.57) | **3.17 (1)*** | -1.17 (0.40) |  |
| Natural Vegetation | 0.26 (0.62) |  | **0.25 (0.66)** | **-2.23 (1)** |
| Residential | 2.05 (0.49) | **6.48 (1)*** | **1.34 (0.70)** | **0.69 (1)*** |
| Soil / Desert | 2.45 (0.49) |  | **1.20 (0.94)** |  |
| Water | 2.01 (0.36) | **5.04 (1)*** | 3.41 (0.22) | 1.07 (0.13) |

Supplemental Table 3: Conditional beta estimates from model averaging for individual species abundance during the spring. The relative importance of each variable is displayed next to the standardized beta estimate in parentheses. The total number of models with 2 DAIC of the top model are displayed before the species name. The maximum relative importance value calculated based on the random null model (see methods) was 0.63. All variables with a relative importance value above 0.63 are bolded for emphasis. Asterisks indicate the standardized beta estimate’s 95% confidence intervals do not overlap with zero.
