# Supplementary material for: No fry zones: How restaurant distribution and abundance influence avian communities in the Phoenix, AZ metropolitan area
Source: PLoS One. 2022 Oct 19;17(10):e0269334. doi: 10.1371/journal.pone.0269334 (PMC9581420; doi:10.1371/journal.pone.0269334)
Supplement: S6 Table — Below, relative importance are the standardized conditional beta estimates with upper and lower 95% confidence intervals for each variable based on the variable’s relative importance across all top models. The top nine models are also displayed below with + indicating the variable is included in the model. Our randomized null model contained variables with estimated relative importance of 0.65, thus variables with a relative importance above 0.65 likely have meaningful predictive power. The variables are listed by relative importance. (DOCX) [file pone.0269334.s008.docx]

Supplemental Table 6: Relative importance of variables within the top models (DAIC <2) for estimates of winter species richness by site. Below, relative importance are the standardized conditional beta estimates with upper and lower 95% confidence intervals for each variable based on the variable’s relative importance across all top models. The top nine models are also displayed below with + indicating the variable is included in the model. Our randomized null model contained variables with estimated relative importance of 0.65, thus variables with a relative importance above 0.65 likely have meaningful predictive power. The variables are listed by relative importance.

|  | Businesses | Highly Developed | Restaurants | Year | Natural Vegetation | Cultivated Vegetation | Water | Residential | Soil / Desert | Cropland |
| --- | --- | --- | --- | --- | --- | --- | --- | --- | --- | --- |
| Relative Importance | 1 | 1 | 1 | 1 | 0.89 | 0.80 | 0.60 | 0.58 | 0.43 | 0.34 |
| Conditional Beta Estimates | 0.10 (0.07 \| 0.13) | 0.45 (-0.92 \| 1.83) | -0.07 (-0.09 \| -0.05) | 0.05 (0.04 \| 0.06) | 0.18 (-0.41 \| 0.77) | 0.02 (-0.04 \| 0.08) | 0.12 (-0.18 \| 0.42) | 0.17 (-0.82 \| 1.16) | 0.28 (-0.30 \| 0.86) | 0.20 (-0.16 \| 0.56) |
| Model 1 | + | + | + | + | + | + | + |  |  |  |
| Model 2 | + | + | + | + | + | + | + | + |  |  |
| Model 3 | + | + | + | + | + |  |  | + | + | + |
| Model 4 | + | + | + | + |  | + |  | + | + | + |
| Model 5 | + | + | + | + | + | + | + | + | + | + |
| Model 6 | + | + | + | + | + | + | + |  | + |  |
| Model 7 | + | + | + | + | + | + |  |  |  |  |
| Model 8 | + | + | + | + | + |  | + |  |  |  |
| Model 9 | + | + | + | + | + | + |  | + | + |  |
