# Supplementary material for: No fry zones: How restaurant distribution and abundance influence avian communities in the Phoenix, AZ metropolitan area
Source: PLoS One. 2022 Oct 19;17(10):e0269334. doi: 10.1371/journal.pone.0269334 (PMC9581420; doi:10.1371/journal.pone.0269334)
Supplement: S7 Table — Below, relative importance are the standardized conditional beta estimates with upper and lower 95% confidence intervals for each variable. The top two models are displayed below with + indicating the variable is included in the model. Variables are listed by relative importance. Our randomized null model contained variables with estimated relative importance of 0.71, thus variables with a relative importance above 0.71 likely have meaningful predictive power. (DOCX) [file pone.0269334.s009.docx]

Supplemental Table 7: Relative importance of variables within the top models (DAIC <2) for estimates of spring species richness by site. Below, relative importance are the standardized conditional beta estimates with upper and lower 95% confidence intervals for each variable. The top two models are displayed below with + indicating the variable is included in the model. Variables are listed by relative importance. Our randomized null model contained variables with estimated relative importance of 0.71, thus variables with a relative importance above 0.71 likely have meaningful predictive power.

|  | Businesses | Cropland | Cultivated Vegetation | | Highly Developed | Natural Vegetation | Residential | Soil / Desert | Water | | Year | Restaurant Count | | |
| --- | --- | --- | --- | --- | --- | --- | --- | --- | --- | --- | --- | --- | --- | --- |
| Relative Importance | 1 | 1 | | 1 | 1 | 1 | 1 | 1 | 1 | 1 | | | 0.24 |  |
| Conditional Beta Estimates | 0.17 (0. 14 \| 0.21) | 2.70 (2.48 \| 2.92) | | 0.96 (0.81 \| 1.21) | 2.31 (2.03 \| 2.59) | 0.67 (0.54 \| 0.80) | 5.48 (4.92 \| 6.04) | 6.11 (5.27 \| 6.95) | 0.32 (0.26 \| 0.40) | -0.35 (-0.40 \| -0.30) | | | 0.01 (-0.01 \| 0.03) |  |
| Model 1 | + | + | | + | + | + | + | + | + | + | | |  |  |
| Model 2 | + | + | | + | + | + | + | + | + | + | | | + |  |
