# Supplementary material for: No fry zones: How restaurant distribution and abundance influence avian communities in the Phoenix, AZ metropolitan area
Source: PLoS One. 2022 Oct 19;17(10):e0269334. doi: 10.1371/journal.pone.0269334 (PMC9581420; doi:10.1371/journal.pone.0269334)
Supplement: S9 Table — No models were within 2 DAIC of the top model. Below, relative importance are the standardized conditional beta estimates for each variable based on the variable’s relative importance across all top models. 95% confidence intervals are shown for each beta estimate in parathesis. The top model is displayed below with + indicating the variable is included in the model. Our randomized null model contained variables with estimated relative importance of 0.73, thus variables with a relative importance above 0.73 likely have meaningful predictive power. The variables are listed alphabetically. (DOCX) [file pone.0269334.s011.docx]

Supplemental Table 9: Relative importance of variables within the top models (DAIC <2) for estimates of spring species abundance by site. No models were within 2 DAIC of the top model. Below, relative importance are the standardized conditional beta estimates for each variable based on the variable’s relative importance across all top models. 95% confidence intervals are shown for each beta estimate in parathesis. The top model is displayed below with + indicating the variable is included in the model. Our randomized null model contained variables with estimated relative importance of 0.73, thus variables with a relative importance above 0.73 likely have meaningful predictive power. The variables are listed alphabetically.

|  | Businesses | Cropland | Cultivated Vegetation | | Highly Developed | Natural Vegetation | Residential | Restaurants | Soil / Desert | Water | | Year |  |
| --- | --- | --- | --- | --- | --- | --- | --- | --- | --- | --- | --- | --- | --- |
| Relative Importance | 1 | 1 | | 1 | 1 | 1 | 1 | 1 | 1 | 1 | 1 | | |
| Conditional Beta Estimates | 0.05 (0.03 \| 0.06) | 2.49 (0.32 \| 4.17) | | 0.90 (0.30 \| 1.50) | 2.09 (0.76 \| 3.42) | 0.61 (0.28 \| 0.93) | 4.98 (1.54 \| 8.32) | 0.06 (0.05 \| 0.07) | 5.73 (1.81 \| 9.65) | 0.28 (0.08 \| 0.48) | -0.15 (-0.17 \|-0.13) | | |
| Model 1 | + | + | | + | + | + | + | + | + | + | + | | |
