# Supplementary material for: No fry zones: How restaurant distribution and abundance influence avian communities in the Phoenix, AZ metropolitan area
Source: PLoS One. 2022 Oct 19;17(10):e0269334. doi: 10.1371/journal.pone.0269334 (PMC9581420; doi:10.1371/journal.pone.0269334)
Supplement: S10 Table — Below, relative importance are the standardized conditional beta estimates for each variable based on the variable’s relative importance across all top models. 95% confidence intervals are shown for each beta estimate in parathesis. The top nine models are displayed below with + indicating the variable is included in the model. Our randomized null model contained variables with estimated relative importance of 0.38, thus variables with a relative importance above 0.38 likely have meaningful predictive power. The variables are listed by relative importance. (DOCX) [file pone.0269334.s012.docx]

Supplemental Table 10: Relative importance of variables within the top models (DAIC <2, 17 models) for assessing community dissimilarity during the winter season. Below, relative importance are the standardized conditional beta estimates for each variable based on the variable’s relative importance across all top models. 95% confidence intervals are shown for each beta estimate in parathesis. The top nine models are displayed below with + indicating the variable is included in the model. Our randomized null model contained variables with estimated relative importance of 0.38, thus variables with a relative importance above 0.38 likely have meaningful predictive power. The variables are listed by relative importance.

|  | Restaurant | Natural Vegetation | Highly Developed | Residential | Businesses | Year | Cultivated Vegetation | Cropland | Soil/ Desert | Water |
| --- | --- | --- | --- | --- | --- | --- | --- | --- | --- | --- |
| Relative Importance | 0.92 | 0.81 | 0.48 | 0.47 | 0.47 | 0.43 | 0.22 | 0.19 | 0.19 | 0.12 |
| Conditional Beta Estimates | -0.05 (-0.08 \| -0.03) | 0.51 (0.36 \| 0.65) | -0.43 (-0.67 \| -0.18) | -0.17 (-0.34 \| 0.01) | -0.01 (-0.02 \| 0.00) | -1.12 (-2.18 \| 0.06) | 0.05 (0.02 \|0.08) | -0.37 (-0.48 \| -0.26) | -0.38 (-0.49 \| -0.027) | 0.18 (-0.02 \| 0.38) |
| Model 1 | + | + |  |  |  | + |  |  |  |  |
| Model 2 | + | + |  |  |  |  |  |  |  |  |
| Model 3 | + |  | + | + | + |  |  | + | + |  |
| Model 4 | + | + | + | + | + | + | + |  |  |  |
| Model 5 | + | + | + | + | + |  | + |  |  |  |
| Model 6 | + | + |  |  |  |  |  | + |  | + |
| Model 7 | + |  | + | + | + | + |  |  | + |  |
| Model 8 | + | + |  |  |  | + | + |  |  |  |
| Model 9 | + | + |  |  |  | + |  |  |  | + |
| Model 10 |  | + | + |  |  |  |  |  |  |  |
| Model 11 | + | + |  | + |  | + |  |  |  |  |
| Model 12 | + | + |  | + |  |  |  |  |  |  |
| Model 13 | + | + | + |  |  |  |  |  |  |  |
| Model 14 | + | + |  |  | + | + |  |  |  |  |
| Model 15 | + | + | + | + | + |  |  |  |  |  |
| Model 16 |  |  | + | + | + |  |  | + |  |  |
| Model 17 | + | + |  |  | + |  |  |  |  |  |
